# LF-15 & T7, Synthetic Peptides Derived from Tumstatin, Attenuate Aspects of Airway Remodelling in a Murine Model of Chronic OVA-Induced Allergic Airway Disease

**DOI:** 10.1371/journal.pone.0085655

**Published:** 2014-01-15

**Authors:** Karryn T. Grafton, Lyn M. Moir, Judith L. Black, Nicole G. Hansbro, Philip M. Hansbro, Janette K. Burgess, Brian G. Oliver

**Affiliations:** 1 Woolcock Institute of Medical Research, Sydney, New South Wales, Australia; 2 Discipline of Pharmacology, The University of Sydney, Sydney, New South Wales, Australia; 3 Centre for Asthma and Respiratory Disease and Hunter Medical Research Institute, The University of Newcastle, Newcastle, Australia; National Heart and Lung institute, United Kingdom

## Abstract

**Background:**

Tumstatin is a segment of the collagen-IV protein that is markedly reduced in the airways of asthmatics. Tumstatin can play an important role in the development of airway remodelling associated with asthma due to its anti-angiogenic properties. This study assessed the anti-angiogenic properties of smaller peptides derived from tumstatin, which contain the interface tumstatin uses to interact with the αVβ3 integrin.

**Methods:**

Primary human lung endothelial cells were exposed to the LF-15, T3 and T7 tumstatin-derived peptides and assessed for cell viability and tube formation *in vitro*. The impact of the anti-angiogenic properties on airways hyperresponsiveness (AHR) was then examined using a murine model of chronic OVA-induced allergic airways disease.

**Results:**

The LF-15 and T7 peptides significantly reduced endothelial cell viability and attenuated tube formation *in vitro*. Mice exposed to OVA+ LF-15 or OVA+T7 also had reduced total lung vascularity and AHR was attenuated compared to mice exposed to OVA alone. T3 peptides reduced cell viability but had no effect on any other parameters.

**Conclusion:**

The LF-15 and T7 peptides may be appropriate candidates for use as novel pharmacotherapies due to their small size and anti-angiogenic properties observed *in vitro* and *in vivo*.

## Introduction

Asthma is a chronic respiratory disease affecting over 300 million people worldwide. One of the most consistent and striking findings in asthma is the structural remodelling of the airway walls, which can contribute to the physiological changes in lung function observed in asthma [1]. Characteristic changes of airway remodelling include epithelial dysplasia, increased bulk of the extracellular matrix and airway smooth muscle layer, and an increased number of blood vessels in the lamina propria and adventitia of the airway wall [2]. Since blood vessels play an important role in the supply of nutrients and oxygen to tissues and the removal of metabolic waste products, the vascularity of the airway wall may be a crucial determinant in airway remodelling.

Angiogenesis is also likely to contribute to the chronic inflammation which occurs in the asthmatic lung. Indeed, angiogenesis and inflammation are two interrelated processes. Influx of inflammatory cells into the airway wall and lumen from the systemic circulation occurs via extravasation across activated endothelial cells. This in turn can promote hypoxia of the inflamed tissue, which then provides the stimulus for further angiogenesis. The greater number of blood vessels provides the potential for increased production of inflammatory mediators and an increased surface area of activated endothelium, thus adding to the inflammatory burden [3].

In humans with asthma the (indirect) relationship between inflammation and airways hyperresponsiveness (AHR) is complex and multifactorial, and likely is different in the different endotypes of asthma. However it has been shown that treatment with only corticosteroids reduces AHR to provocative stimuli [4]. In animal models of allergic airways disease, a proxy for asthma, where is it possible to genetically delete or overexpress a number of mediators, several inflammatory mediators have been found to be necessary for the development of AHR. Given the relationship between angiogenesis, inflammation and AHR, it is reasonable to hypothesise that reducing blood vessel number would also reduce inflammation and AHR.

Angiogenesis is a complex biological process controlled by the balance of pro- and anti-angiogenic factors [5]. We have previously compared a number of anti-angiogenic factors between asthmatic and non-asthmatic airways and found that tumstatin, the NC1 domain of the collagen IV α3 chain, was markedly reduced in the airways of people with asthma [5]. Importantly, increasing tumstatin expression in a murine model of allergic airways disease (AAD) inhibited angiogenesis, pulmonary inflammation and AHR) [5]. These findings suggest that tumstatin, delivered directly to the airways, may have therapeutic potential in asthma. A key step in this development is to identify components of the native tumstatin molecule which can be easily synthesised. Several tumstatin-derived anti-angiogenic peptides have been described, the most potent of which are the overlapping peptides T3 (69–88 amino acids) and T7 (74–98 amino acids) [6].

Eikesdal *et al* identified an interactive interface for the T7 peptide on the αVβ3 integrin involving the amino acids leucine, valine and aspartic acid and showed that this region was essential for T7′s anti-angiogenic activity [6]. Interestingly, these amino acids are found in this overlapping region of the T3 and T7 peptides [6]. Given the prediction that the active anti-angiogenic site is located in this region, we hypothesised that a novel peptide consisting only of the overlapping region, which we termed LF-15, would possess the anti-angiogenic properties of both T3 and T7 and may also have greater efficacy. We also hypothesised that the LF-15 peptide would retain tumstatin's ability to inhibit AHR and pulmonary inflammation. We envisage that LF-15 may be an attractive therapeutic candidate due to its smaller size, potentially increasing its bioavailability whilst reducing manufacturing costs compared to tumstatin [7]. To this end, this study aimed to investigate LF-15′s anti-angiogenic capacity in comparison to T3 and T7 using a range of *in-vitro* angiogenic assays, and a murine model of chronic AAD in which we evaluated the ability of LF-15 to inhibit angiogenesis, inflammation and AHR.

## Methods

### Ethics statement

Human airway tissue was obtained from explanted and resected lungs and post mortem organ donors with ethical approval from The University of Sydney and participating hospitals (Concord Repatriation General Hospital, Sydney South West Area Health Service and Royal Price Alfred Hospital) for sample collection. All volunteers, or their next of kin, provided written informed consent. Ethical approval for experiments on animals was given by the Animal Care and Ethics Committee of the University of Newcastle.

### Endothelial cells

Primary endothelial cells were cultured from explanted lungs, as previously described [5], and grown in tissue culture flasks pre-coated with 0.2% (w/v) gelatine (Sigma-Aldrich, St Louis, USA) which contained Ham's F-12 culture medium (Invitrogen, Carlsbad, USA), supplemented with 15 mM HEPES (Sigma-Aldrich), 0.035 mg/ml Endothelial Cell Growth Supplement (ECGS) (Sigma-Aldrich), 20 U/ml Heparin (Sigma-Aldrich), 20% FBS (JRH Biosciences, Melbourne, Australia) and antibiotics (1 U/ml penicillin, 1µg/ml streptomycin and 250 ng/ml amphotericin) (Invitrogen). Cells between passages 3-6 were used for all experiments. Human Umbilical Vein Endothelial Cells (HuVEC) were purchased from the American Type Culture Collection, VA USA (Catalogue No. CRL-1730).

### Stimulation with LF-15, T3 and T7 peptides

**Figure 1:**
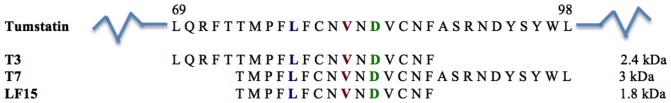
Amino acids sequences of the T3 (2.4 kDa), T7 (3 kDa) and LF15 (1.8 kDa) peptides in comparison to tumstatin (28 kDa). LF15 (amino acids 74–88) encompasses the overlapping region shared by the T3 (69–88) and T7 (74–98) peptides. Peptide integrin binding sites involve the amino acids L (78, Leucine), V (82, Valine) and D (84, Aspartic acid).

Custom-made T3 (69–88) and LF-15 (74–88) peptides were obtained from GL Biochem Ltd, Shanghai, China, and T7 peptides were obtained from Proteomics International Pty Ltd, Perth, Australia (see figure 1). Endothelial cells were seeded into 96 well plates at a density of 1×10^4^ cells/cm^2^ in HAMS Nutrient F-12 (JRH Biosciences) containing 20% FBS and 35µg/ml ECGS (BD Biosciences) and grown for a period of 24 hours at 37°C in 5% CO_2_. Cells were then quiesced in F12 supplemented with 0.1% BSA for 24 hours. Cells were then treated with 4.5µM LF-15, T3 or T7 peptides or vehicle control (acetonitrile and TFA) in growth medium and incubated at 37°C in 5% CO_2_. Peptides were replenished after 24, 72 and 144 hours.

### MTT assay

The effect of the T3, T7 and LF-15 peptides on cell viability was assessed using a commercially available 3-(4,5-Dimethylthiazol-2-yl)-2,5-Diphenyltetrazolium Bromide (MTT) assay (Sigma-Aldrich). To assess for cytotoxicity using the MTT assay, cells were grown and treated in 96 well plates as per experimental protocol before the addition of 10µl sterile MTT (5 mg/ml) per well six hours before the experiment was due to conclude on Days 3, 7 and 9. After six hours, 100µl of 10% sodium dodecyl sulphate (SDS) (wt/vol) (Amresco, Solon, USA) in 0.01 M HCL (ThermoFisher Scientific, VIC, Australia) was added to each well and plates were left to incubate overnight at 37°C in 5% CO_2_. Absorbance was measured at 570 nm with a background reading of 690 nm using a Wallac-1000 plate reader and software (PerkinElmer, Massachusetts, USA).

### Tube formation assay

The BD BioCoat Angiogenesis system - endothelial cell tube formation - Matrigel assay (BD Biosciences, MA USA) was used to assess the anti-angiogenic activity of the T3, T7 and LF-15 peptides. Endothelial cells were seeded onto a 96 well plate at 4×10^5^ cells/ml in F-12 media with ECGS containing 10% FBS, with 100 ng/ml angiopoietin-1 (R&D systems, Minneapolis, USA) 300 ng/ml ephrine B2 (R&D systems) and 100 ng/ml VEGF (R&D systems). The T3, T7 and LF-15 peptides or corresponding vehicle controls, were added to triplicate wells at a concentration of 4.5µM. After 18 hours incubation at 37°C in 5% CO_2_, tube formation was visualised using an inverted light microscope. Images were taken using a digital camera (Olympus *CAMEDIA* C-4000) and the total number of tubes per well were counted manually.

### Chronic OVA-induced AAD

A murine model of chronic OVA-induced AAD was generated as previously described [5], [8], [9]. Balb/C mice were administered tumstatin peptides diluted in PBS at a concentration of 30 ng or 300 ng per 20 g body weight or PBS alone once a day from days 25 to 115. Specific airway resistance to inhaled methacholine (3, 5, and 10 mg/ml) was calculated on Day 115 after which the mice were sacrificed, and the left lungs were removed and inflated with 30% formalin/PBS for 10 minutes [5]. Lungs were then fixed in 4% formaldehyde solution for immunohistological analysis.

### Histology

The degree of airway vascularity and extent of inflammation within the airways was assessed by histology. 5µm sections of lung specimens were stained with Harris haematoxylin (Sigma-Aldrich), washed using running tap water, then placed in acid alcohol (Sigma-Aldrich) to differentiate. Sections were then washed in running tap water and blued in Scott's tap water (Sigma-Aldrich). After rinsing in tap water, sections were stained with eosin (Sigma-Aldrich) then dehydrated through graded alcohols, cleared in xylene (MP Biomedicals Inc, Santa Ana, USA) and mounted using Faramount aqueous mounting medium (DakoCytomation, Glostrup, Denmark) and coverslipped. Sections were graded as per custom histopathology criteria designed by Horvat *et al* [10].

#### Endoglin/CD105 Immunohistochemistry

Immunohistochemistry was used to assess the effect of the peptides T3, T7 and LF15 on the number of blood vessels present in lung tissue. Briefly, slides were treated with peroxidase blocking reagent (DakoCytomation) for 30 mins then washed in x2 changes of dH_2_O. Slides were then blocked with blocking serum (VECTASTAIN Elite ABC KIT, Vector laboratories). Primary antibody (goat anti-mouse CD105, R&D Systems) or isotype control at a concentration of 15µg/ml was added to each section and incubated for 30 mins at room temp. After washing with x2 changes of T-PBS, slides were incubated with biotinylated secondary antibody (VECTASTAIN Elite ABC KIT, Vector laboratories). Slides were washed in T-PBS and incubated with Vectastain elite ABC reagent (VECTASTAIN Elite ABC KIT, Vector laboratories) for 30 mins. Next, slides were washed in T-PBS and incubated with DAB (DakoCytomation) for 10 mins. Slides were washed with dH_2_O, dehydrated and mounted.

### Statistical analysis

All data were analysed using GraphPad PRISM 5.0 (GraphPad, La Jolla, USA). Statistical analysis was performed using one way and two way analysis of variance (ANOVA) with appropriate post-hoc tests depending on Gaussian distribution of data. Differences were considered significant when p<0.05.

## Results

### LF-15, T3 and T7 decrease primary lung endothelial cell viability

**Figure 2:**
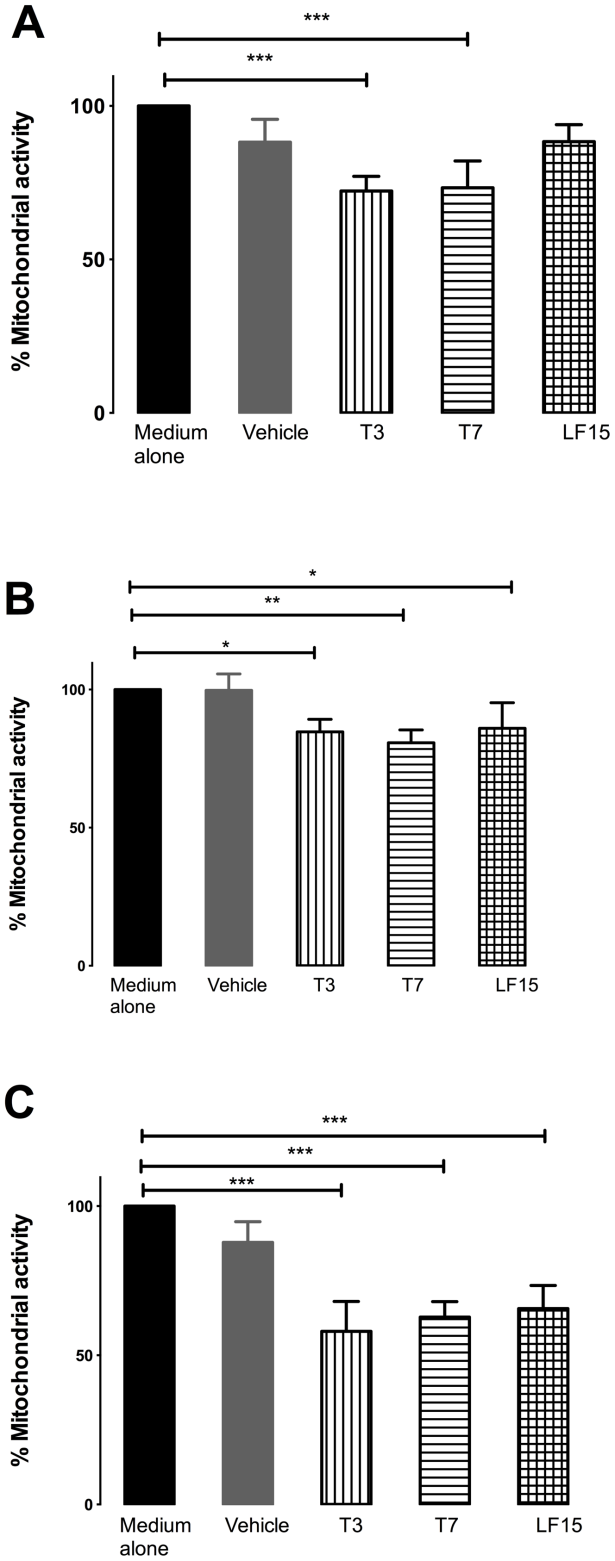
T3, T7 and LF-15 inhibit mitochondrial function in primary lung endothelial cells. Cells were treated with T3 or T7 or LF-15 peptides for 3 (A), 7 (B) and 9 (C) days before mitochondrial function assessed using the MMT assay. *p<0.05,**p<0.01,***p<0.001 vs medium alone, n = 8.

Primary lung endothelial cell viability following 3, 7 and 9 days treatment with the LF-15, T3 and T7 tumstatin-derived peptides was assessed using the MTT assay. All three peptides significantly reduced cell viability at all time points (Figure 2; p<0.05 vs medium alone), with the exception of LF-15 at Day 3. At Days 7 and 9 all peptides had reduced cell viability to a similar extent (statistical analysis not performed).

### LF-15 and T7, but not T3, inhibit endothelial cell tube formation

**Figure 3:**
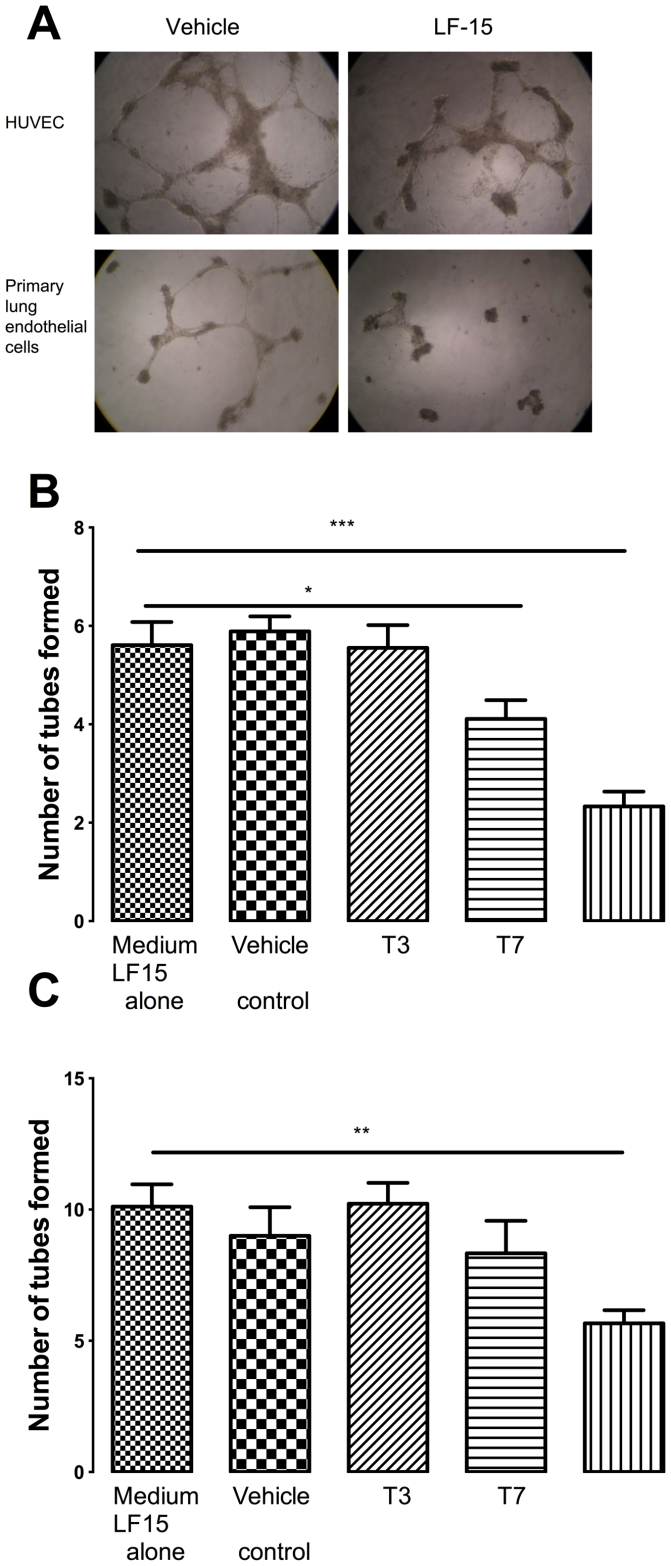
LF-15 decreased endothelial tube formation. LF-15 decreased endothelial tube by primary lung endothelial cells (B) and HUVECs (C). Cells were treated with T3 or T7 or LF-15 peptides for 18 hours before the extent of tube formation was assessed by light microscopy. Image (A) is a representative image of tube formation following treatment with the LF-15 peptide. *p<0.05,**p<0.01, ***p<0.001 vs medium alone, n = 6.

The ability of LF-15, T3, and T7 to inhibit primary lung endothelial cell tube formation was evaluated using an *in-vitro* angiogenesis assay (Figure 3A). Treatment of primary lung endothelial cells with LF-15 or T7 peptides for 18 hours significantly decreased the number of tubes formed by 18% and 53% respectively (Figure 3B; p<0.05 vs control). In contrast, treatment of primary lung endothelial cells with the T3 peptide did not affect tube formation.

Since we observed differential effects of LF-15 and T7 in comparison to T3, in terms of inhibiting primary pulmonary endothelial cell tube formation, we repeated the experiments using HuVECs to investigate if this differential efficacy was restricted to lung endothelial cells. In this set of experiments, only LF-15 affected tube formation in HuVECs, whereby tube formation was significantly reduced by 44% (Figure 3C; p<0.05 vs control). T7 and T3 peptides had no effect on HuVec tube formation.

### LF-15 and T7, but not T3, suppress AHR in chronic OVA-induced AAD

A murine model of chronic OVA-induced AAD was used to examine the anti-angiogenic properties of the peptides *in vivo*.

**Figure 4:**
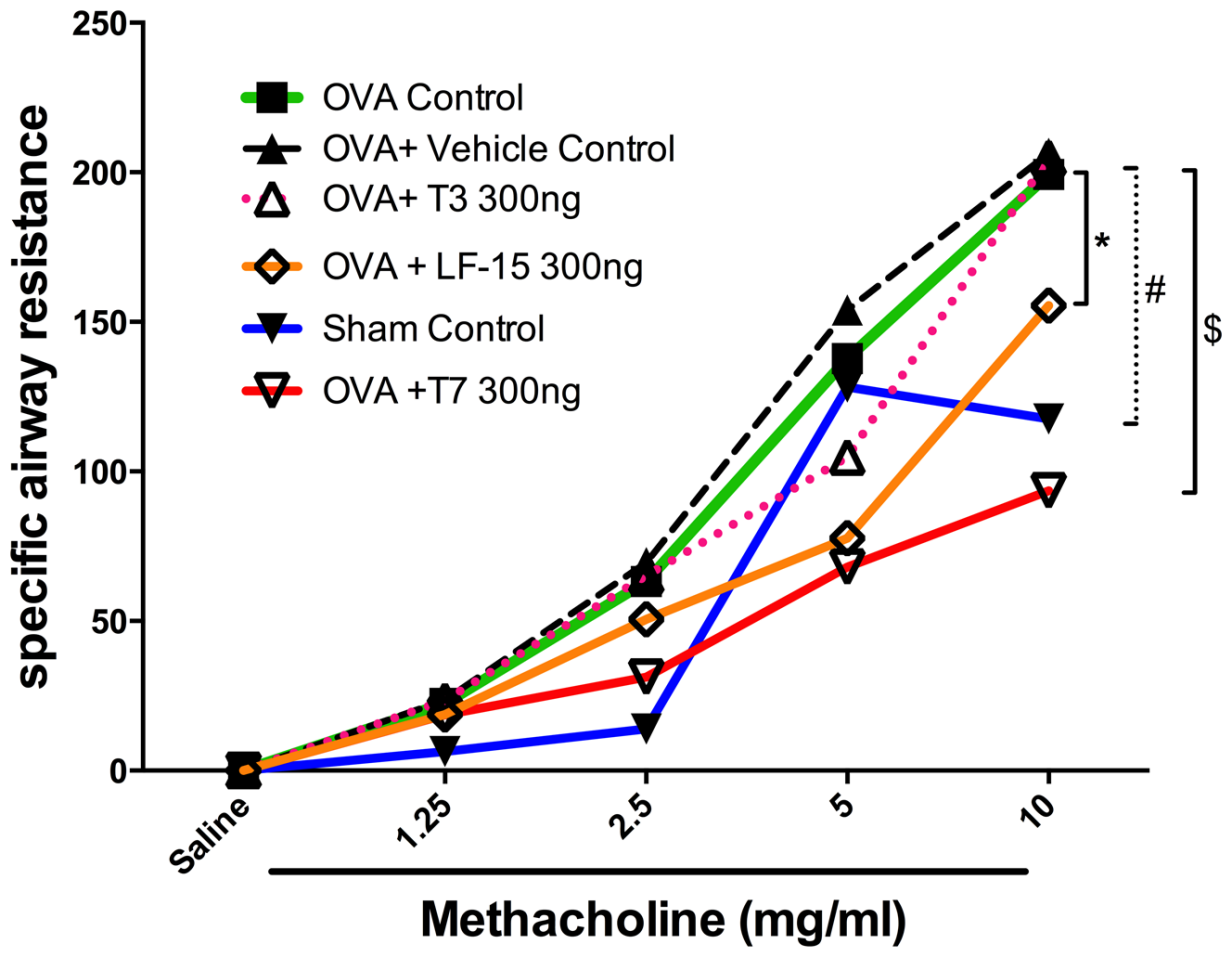
LF-15 and T7 attenuate OVA-induced airways resistance in a murine model of allergic airways disease. OVA sensitised mice were exposed to the T3, T7 and LF-15 between Day 25 and Day 115 before specific airway resistance following methacholine challenge was assessed. *p<0.05 OVA+LF-15 vs OVA+ Vehicle control, ^#^p<0.05 OVA+Vehicle control vs Sham control, $p<0.05 OVA+T7 vs OVA+ Vehicle control, n = 6.

BALB/c mice that were chronically exposed to OVA +/− vehicle demonstrated a significantly greater increase in airway resistance to methacholine at 5 and 10 mg/ml than saline-treated mice (Figure 4; p<0.01 vs sham). In the mice treated with OVA+ LF-15 (300 ng/ml) airway resistance was significantly lower than those treated with only OVA at 5 and 10 mg/ml methacholine (Figure 4; p<0.05 vs OVA + vehicle control). Similarly, in the mice treated with OVA+ T7 (300 ng/ml) airway resistance was significantly lower than those treated with only OVA at 5 and 10 mg/ml methacholine (Figure 4; p<0.05 vs OVA + vehicle control). In contrast, the T3 peptide had no effect on airway resistance to methacholine.

### LF-15 and T7, but not T3, decrease lung blood vessel area in chronic OVA-induced AAD

**Figure 5:**
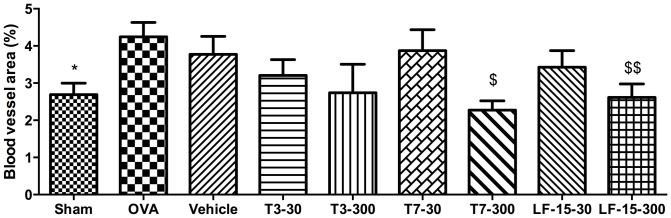
LF-15 and T7 attenuate OVA-induced changes in lung blood vessel area in a murine model of allergic airways disease. OVA sensitised mice were exposed to the T3, T7 and LF-15 between Day 25 and Day 115 before mice were sacrificed and the blood vessel area in the lungs was determined by counting CD105 positive blood vessels.*p<0.05 Sham vs OVA, ^$^p<0.05 vs OVA, ^$$^p<0.01 vs OVA, n = 8.

The development of AAD in BALB/c mice treated with OVA coincided with a significant increase in the total lung area occupied by blood vessels compared to saline-treated mice which did not develop AAD (4.2% vs 2.7% respectively) (Figure 5; p<0.05 vs sham).

OVA-treated mice that were also treated with LF-15 or T7 had a significantly lower total lung area occupied by blood vessels (Figure 5; p<0.05 vs OVA). The total lung area of blood vessels in these mice was similar to those which were not exposed to OVA. In contrast, mice treated with OVA+T3 had the same total lung area occupied by blood vessels as mice treated with OVA alone (Figure 5).

### LF-15 and T7 decrease pulmonary inflammation in chronic OVA-induced AAD

**Figure 6:**
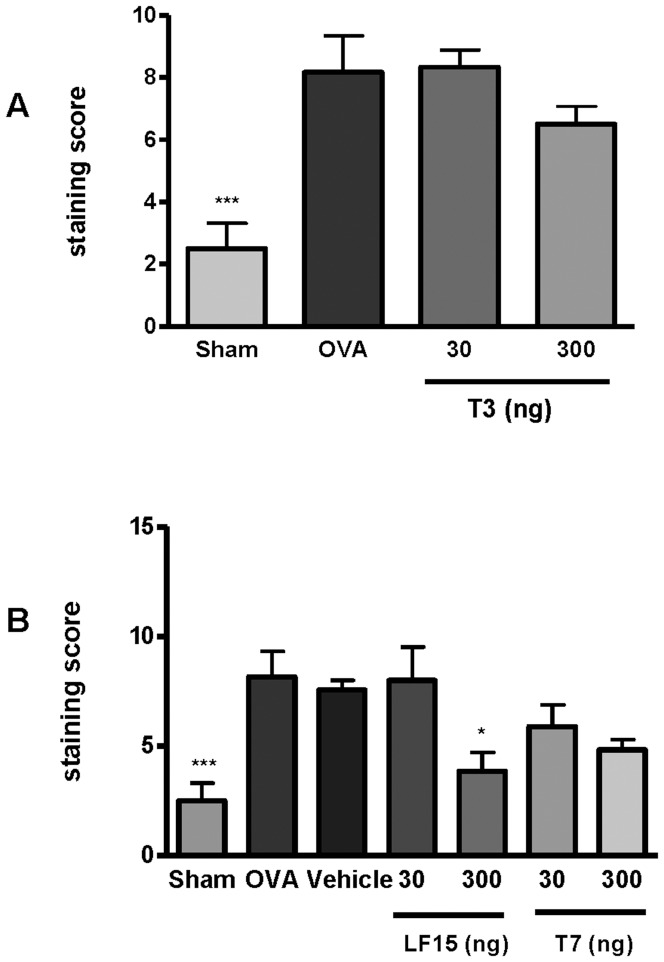
LF-15 attenuates OVA-induced pulmonary inflammation in a murine model of allergic airways disease. OVA sensitised mice were exposed to the T3, T7 and LF-15 between Day 25 and Day 115 before mice were sacrificed and the extent of pulmonary inflammation was determined by H&E staining. ***p<0.05 Sham vs OVA, ^*^p<0.05 LF-15 300 ng vs OVA, n = 8.

In the murine model of AAD, chronic treatment with OVA significantly increased pulmonary inflammation as measured by a scoring system developed to quantify inflammatory cells inflammation in H&E stained sections [10] (Figure 5; p<0.001 vs sham). When OVA-treated mice were also exposed to LF-15, the extent of pulmonary inflammation was significantly decreased (Figure 6; p<0.05 vs OVA). In comparison, treatment with T3 or T7 peptides had no effect on pulmonary inflammation.

## Discussion

This study has demonstrated for the first time using a murine model of chronic OVA-induced AAD that the novel LF-15 peptide, derived from the native tumstatin molecule, can inhibit endothelial cell viability and tube formation *in vitro*, and suppress pulmonary inflammation, angiogenesis and AHR *in vivo*. Therefore, LF-15 may have therapeutic potential in asthma. Furthermore, this study also demonstrated that LF-15 appeared to be more biologically active and was as good, if not superior, to T3 and T7 at attenuating angiogenesis and aspects of AAD. This may be due to LF-15 encompassing properties of both T3 and T7, as the LF-15 peptide is comprised of the overlapping area between the T3 and T7 peptides, which contains the interface that interacts with integrins [6].

We used a range of contemporary angiogenic *in-vitro* assays in addition to evaluating the efficacy of the peptides in an *in-vivo* model of AAD. We found that all three peptides reduced endothelial cell viability at days 7 and 9. However at day 3, LF-15 did not affect viability, whilst T3 and T7 did. We are not certain why this might be the case, but potentially LF-15 might have a slower mechanism of action or reduced potency in this assay. When we assessed the ability of the peptides to inhibit endothelial cell tube formation, only LF-15 and T7 inhibited tube formation. This suggests that the attenuation of tube formation was not purely a result of reduced cell viability. Furthermore, in our *in-vivo* model of AAD, LF-15 and T7 inhibited the development of AHR, blood vessel formation and pulmonary inflammation but the T3 peptide did not affect any features of AAD tested. Therefore it may be reasonable to conclude that the mechanisms by which these peptides inhibited the development of these features of AAD is more complex than solely a reduction in endothelial cell viability.

The concentrations of peptides used *in-vitro* in this study were based on the effective concentrations used by Maeshima *et al.*, 2001 [11], and our previous study [5], which showed that the effective concentrations of T3 and T7 were between 1-4.5 µM. As the interactive interface is the same for all three peptides [6], it is unlikely that potency *per se* accounts for the differences observed between T3 compared to LF-15 and T7. However, it is worth noting that the effective concentrations of T3 and T7 were based upon inhibition of cellular viability [5], [11], which was induced by all three peptides in the current study. Theoretical mapping of the T7 peptide and the αVβ3 integrin predicted binding to occur in the region which does not overlap with the T3 peptide (i.e. LF-15) [12]. Thus, it would be expected that T3 and T7 would have different biological activities, as we have observed. However, because LF-15 was as active as the T7 peptide, this suggests that either the region which binds to the αVβ3 integrin is in this region, or the mechanism of action of both LF-15 and T3 is independent of this particular integrin.

We are not certain why the peptides have different biological properties in different assays, but speculate that the T3 peptide contains an inhibitory motif in the region that is not overlapping with T7 and LF-15. Whilst we have no specific data to support this argument, there is good evidence that collagen derived fragments containing as few as three amino acids can have observable biological activity [13].

A murine model of chronic OVA-induced AAD was used to examine the biological activities of the peptides. Whilst acute models of AAD do not demonstrate angiogenesis [11], chronic models are characterised by the development of persistent inflammation, AHR and aspects of airway remodelling that include angiogenesis [3], [6]. These characteristics of chronic models of AAD are consistent with asthma in humans [14], [15]. We previously examined the anti-angiogenic activity of tumstatin *in-vivo* using a chronic OVA-induced model of AAD, and found that the full length protein inhibited angiogenesis, reduced pulmonary inflammation, and improved AHR [5]. These findings are furthered by our current study that demonstrated that LF-15 and T7 also produced these changes. Future studies that directly compare LF-15 and T7 with native tumstatin would be useful in determining the clinical potential of these molecules and paving the path for clinical trials.

The smaller size of the LF-15 peptide may be appealing compared to the full-length tumstatin molecule or the larger T7 peptide for pharmaceutical application. The smaller sized peptide would make for more cost effective production. However clinical trials would be needed to verify if these observations are reproducible in humans. In conclusion, in this study we found that the novel tumstatin-derived peptide LF-15 is more effective than the T3 and T7 peptides in suppressing angiogenesis, pulmonary inflammation and AHR *in-vitro* and *in-vivo*, and therefore may represent a promising therapeutic for the treatment of asthma.
